# High accuracy determination of the thermal properties of supported 2D materials

**DOI:** 10.1038/srep12422

**Published:** 2015-07-16

**Authors:** Jarosław Judek, Arkadiusz P. Gertych, Michał Świniarski, Anna Łapińska, Anna Dużyńska, Mariusz Zdrojek

**Affiliations:** 1 Faculty of Physics, Warsaw University of Technology, Koszykowa 75, 00-662 Warsaw, POLAND

## Abstract

We present a novel approach for the simultaneous determination of the thermal conductivity *κ* and the total interface conductance *g* of supported 2D materials by the enhanced opto-thermal method. We harness the property of the Gaussian laser beam that acts as a heat source, whose size can easily and precisely be controlled. The experimental data for multi-layer graphene and MoS_2_ flakes are supplemented using numerical simulations of the heat distribution in the Si/SiO2/2D material system. The procedure of *κ* and *g* extraction is tested in a statistical approach, demonstrating the high accuracy and repeatability of our method.

2D materials such as graphene or transition metal dichalkogenides (e. g., MoS_2_ and WS_2_) often possess unique mechanical, electrical and optical properties and are therefore foreseen as building blocks for future electronic nano-devices^1^. One of the most significant constraints in the design and fabrication of modern integrated electronic circuits is heat dissipation^2^. Therefore, the knowledge of the phonon and thermal properties, particularly the values of the thermal conductivity *κ* and total interface conductance *g,* is of great importance. A state-of-the-art contactless technique that has been widely and successfully applied in the simultaneous determination of both *κ* and *g* is the opto-thermal method, which employs micro-Raman spectroscopy^3^,^4^,^5^,^6^,^7^,^8^.

To understand the physical basis of the opto-thermal method, one has to recall three facts. First, the solution of the heat diffusion equation describing the temperature distribution in a 2D system depends on the size of the heat source. Second, micro-Raman spectroscopy is a technique that allows for simultaneous local heating of the examined thin flake and probing the local temperature increase. Third, the Raman laser beam spot size focused by the microscope objective on the examined sample depends on its numerical aperture (*NA*). The combination of all of the above enables the extraction of *κ* and *g*; however, there are some serious disadvantages: the lack of confidence about the focus position and, therefore, possible large uncertainty of the beam size; extremely small number of experimental points limited by the number of physically available objectives with different *NA*; difficult automation; and finally, no effect of the heating of the substrate that decreases the heat flux from the examined flake.

In this work, we employ a new approach for the data acquisition and succeeding theoretical analysis that successfully eliminates all of the above weak points. The first three problems are removed by exploiting a property of the laser beam – the precisely defined power distribution in all directions^9^ – and the last one by introducing an enhanced model, including the substrate, which describes most of the realistic nano-devices.

A high accuracy and automatic method for determining *κ* and *g* opens new perspectives for the characterization of both supported and free-standing 2D materials. One of the most attractive perspectives is the possibility of studying the distribution of the thermal properties in the micro-scale in a statistical approach, allowing for the examination of the effects of thermal conductivity variations on heat transfer, correlations between *κ* and *g*, and whether a higher interaction between the 2D material and substrate implies the higher value of the interface conductance and lower value of the thermal conductivity. The above issues are interesting from both the physical and technological point of view, and the method presented here is capable of addressing them. Similar possibilities to opto-thermal method has the time domain thermoreflectance (TDTR)^10^ and magneto-optic Kerr effect thermometry (MOKE)^11^ which also allow for the extraction of the in-plane thermal conductivity and whose resolution enables for *κ* mapping^12^. However, in contrary to the Raman technique, TDTR and MOKE require covering the examined samples by thin metal layer and therefore they are destructive.

## Theory

The temperature distribution *T*(*r*) in a 2D material in the diffusive regime (above room temperature assumed at least for graphene^3^,^13^ and MoS_2_^14^,^15^; however, for the first one, some doubts have recently emerged^16^) in the presence of laser heating can be obtained using the following differential equation:


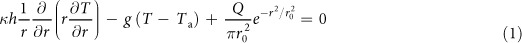


Here, *κ* is the thermal conductivity of the examined thin film characterized by the thickness *h*, and *g* is the total interface conductance that describes the heat transfer from the sample to the substrate of the constant temperature *T*_a_. We can safely neglect the value of the substrate temperature without any loss of generality. After this modification, the meaning of *T*(*r*) changes from the temperature distribution to the temperature increase distribution. The last term in equation (1) describes the heating source and is directly related to the properties of the laser beam; *Q* is the total absorbed laser power, and *r*_0_ is the beam size. Eq. 1 has a physical solution in the form:


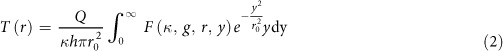


where


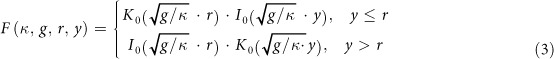


Because the laser beam, which is simultaneously heating the sample and probing the local temperature increase, is spatially distributed, the temperature increase distribution should be averaged to obtain the compatibility with the experimental temperature increase value obtained from Raman measurements (*T*(*r*) →*T*_m_):


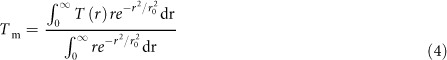



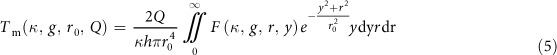


Importantly, the averaged temperature increase expressed in eq. 5 is a function of four parameters. Because the total heating power *Q* (the power of the laser beam multiplied by the absorption) and laser beam size *r*_0_ are usually known or are easy to obtain, the above equation can be used to extract the thermal conductivity and total interface conductance if only two local temperature increase values for different beam spot sizes are known. The typical procedure includes measurements of the derivative of the local temperature increase with respect to the absorbed laser power for two microscope objectives with different *NA* (at the focus *r*_0_ = *M*^2^*λ*/π*NA*, *M*^2^–beam quality factor, and *λ*–laser wavelength) followed by a numerical solution of the system of two nonlinear equations. As previously noted, such an approach is not perfect, but it could be greatly enhanced by utilization of the knowledge of the power distribution of the laser beam in both directions and by the introduction of the substrate to the model.

An useful equation that describes the Gaussian laser beam intensity in the cylindrical coordinates (as a function of the distance from the center axis *r* and the distance from the beam waist *z*_L_) can be written in the following form:


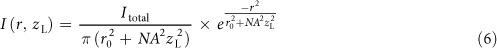


**Figure 1:**
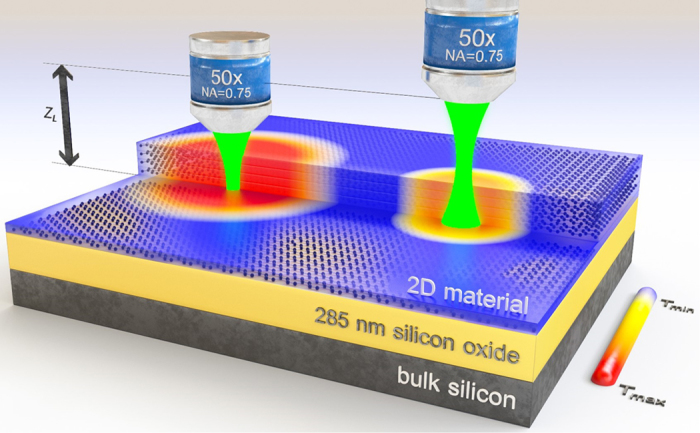
An artistic illustration of the concept of the experiment. Examined 2D material is placed onto the typical Si/SiO_2_ substrate. A Raman laser beam is simultaneously causing and probing the local temperature increase. Depending on the distance of the microscope objective from the sample surface, the beam spot on the sample has a different size, inducing a different temperature increase.

Here, *r*_0_ stands for the minimal beam width in the beam waist and *I*_total_ is the total intensity of the laser beam. In our experiment, to eliminate any unnecessary assumptions, we found the values of the beam parameters ourselves (*r*_0_ = 0.386 μm, *NA* = 0.530, *M*^2^ = 1.25) using the knife-edge technique. These values differ from the usually met values due to the slightly different definition of the intensity dependence in eq. 6. Knowledge of the spatial power distribution of the Raman laser beam is especially useful when combined with the motorized XYZ stage. Setting the plane of the sample at different distances *z*_L_ with respect to the beam waist (in the Z direction, see Fig. 1 for details) allows us to vary the beam size on the sample, eliminating the problem of the uncertainty in the focus position and the small number of experimental points. Additionally, it is very easy to automate.

The introduction of the substrate into the model is more sophisticated and should be preceded by the evaluation of the type of model extension that is required. In this paper, we consider a system equivalent to the one presented in Fig. 1 consisting of the examined thin flake (up to a few nm thick), a silicon oxide dielectric layer (285 nm thick) and a bulk silicon substrate. In all of our experiments, we did not see any sign of heating of the substrate (in the uncertainty limit). Therefore, we include only the dielectric layer in our model, assuming no temperature increase on the Si/SiO_2_ interface. However, if needed, it is possible to add bulk Si in the same manner as the dielectric layer. It is also possible to take into account parasitic heat loss mechanisms like convection and radiation, however, in this paper, in order not to hinder understanding of the main thread we consciously miss this two contributions. The temperature increase distribution *T*(*r*) in 2D material in the diffusive regime over the dielectric layer with the temperature increase distribution *T*_dl_(*r*, *z*) is governed by following a partial differential equation system:


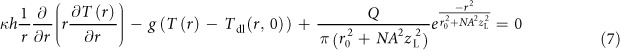



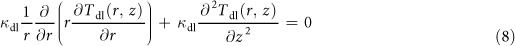


with the following boundary conditions:


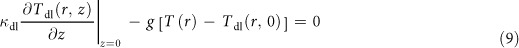



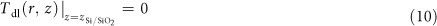


In the above equation, we assumed that at *z* = 0, there is an interface between the examined flake and SiO_2_ dielectric layer^17^ and that *z*_Si/SiO2_ = −285 nm is the interface between the SiO_2_ and bulk silicon. The solution of eqs. 7 and 8 with the boundary condition expressed in eqs. 9 and 10 can be obtained in two ways, analytically and numerically. In this work, we used the second approach; we employed the Finite Element Method implemented in Mathematica software^18^. We note that *z*_L_ describes the laser beam and is a parameter in the eq. 7, whereas *z* is a variable in the sample frame of reference. Moreover, the measured temperature increase is now a function of six parameters: *T*_m_(*κ*, *g*, *z*_L_, *r*_0_, *NA*, and *Q*); the last three parameters are considered to be constant and known, the first two parameters are to be extracted, and *r*_0_ is the independent variable.

**Figure 2:**
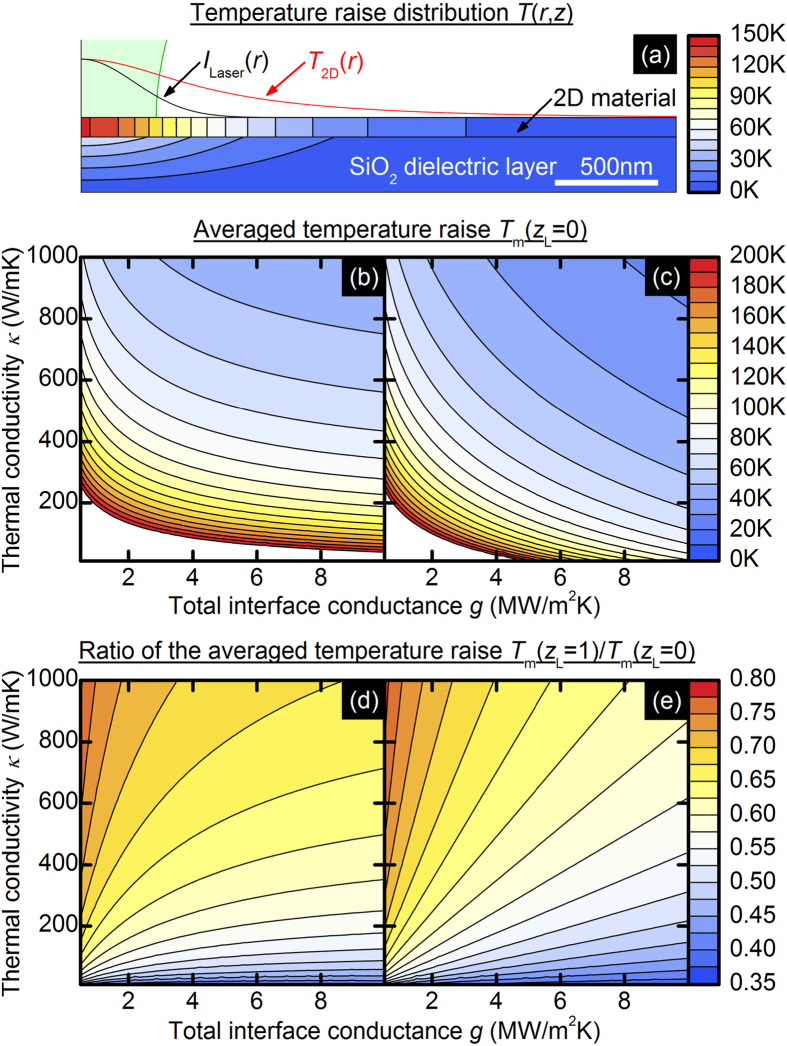
(**a**) Calculated temperature increase distribution *T*(*r*,*z*) in a 5-nm thick layer of 2D material onto a 285-nm thick SiO_2_ substrate under laser light heating. For clarity, the thickness of the first layer in the picture is not to scale. The following are the assumptions used in the model: *κ*_2D_ = 300 W/mK, *κ*_SiO2_ = 1.37 W/mK, *g*_2D/SiO2_ = 3 MW/m^2^K, *Q* = 1 mW, *NA* = 0.530, *r*_0_ = 0.386 μm and *z*_L_ = 0. The averaged value of the temperature increase *T*_m_ calculated for different values of the thermal conductivity and total interface conductance is shown in (**b**) when the effect of the heating of the substrate is included and in (**c**) when this effect is neglected. The ratio of the temperature increase calculated at the focus *T*_m_(*z*_L_ = 0) and 1 μm from the focus *T*_m_(*z*_L_ = 1 μm) for different values of *κ* and *g* is shown in (**d**) when the effect of the heating of the substrate is included and in (**e**) when this effect is neglected.

An exemplary calculation of the temperature increase distribution under laser heating using eqs. 7, 8, 9, 10 is shown in Fig. 2a. The parameters used in this simulation are listed in the caption and are close to the parameter values from the experiment, which is described further. The maximum temperature increase achieved in the 2D material equals 141 K, the average temperature increase is 110 K, and the maximum temperature increase in the SiO_2_ equals 49 K. For comparison, in a model that neglects the heating of the substrate, the maximum temperature increase in thin film equals 120 K and the average temperature increase is 90 K. Higher temperatures in the model with a substrate are quite intuitive because the heated substrate limits the heat flux from the 2D layer, as expressed in the second term in eq. 7. Discontinuity of the temperature values at the interface between the 2D material and substrate are typical for problems with Robin (third kind) boundary conditions (eq. 9).

In Fig. 2b,c, we show the calculated averaged temperature increases (see eq. 4) for different values of *κ* and *g* describing the 2D material in the cases when the effect of the substrate heating is included or neglected. In both cases, the lower interface conductance or thermal conductivity implies a higher temperature increase. However, in Fig. 2b, the temperature increase values are higher, as discussed earlier, and the sensitivity of the *T*_m_ on *g* changes are lower, especially for the higher values of *g*. The last conclusion should also be quite intuitive because the dielectric layer with a much lower *κ* than 2D material acts as another heat barrier.

In Fig. 2d,e, we present calculations of the ratio of the temperature increase for two different laser beam spots when the effect of the substrate heating is included or neglected. The main striking difference between Fig. 2d,e is the loss of sensitivity of the above ratio on the changes of *g* for higher values, which is similar to the difference between Fig. 2b,c. The *T*_m_(*z*_L_ = 1 μm)/*T*_m_(*z*_L_ = 0) ratio reflects the dependence of the temperature increase on the beam size and is related to the “width” of the curve *T*_m_(*z*_L_) with a constant rest of the following parameters: *κ*, *g*, *r*_0_, *NA* and *Q*. Moreover, based on Fig. 2b,d or c,e, one can conveniently graphically estimate the *κ* and *g* values.

## Experimental results and Discussion

We tested our method on two well-known 2D materials, multilayer graphene and multilayer MoS_2_. We intentionally decided to use multilayer structures to focus only on the thermal properties and obey problems that occur in the case of monolayers, optical doping^19^ and degradation upon laser light irradiation^20^,^21^.

**Figure 3:**
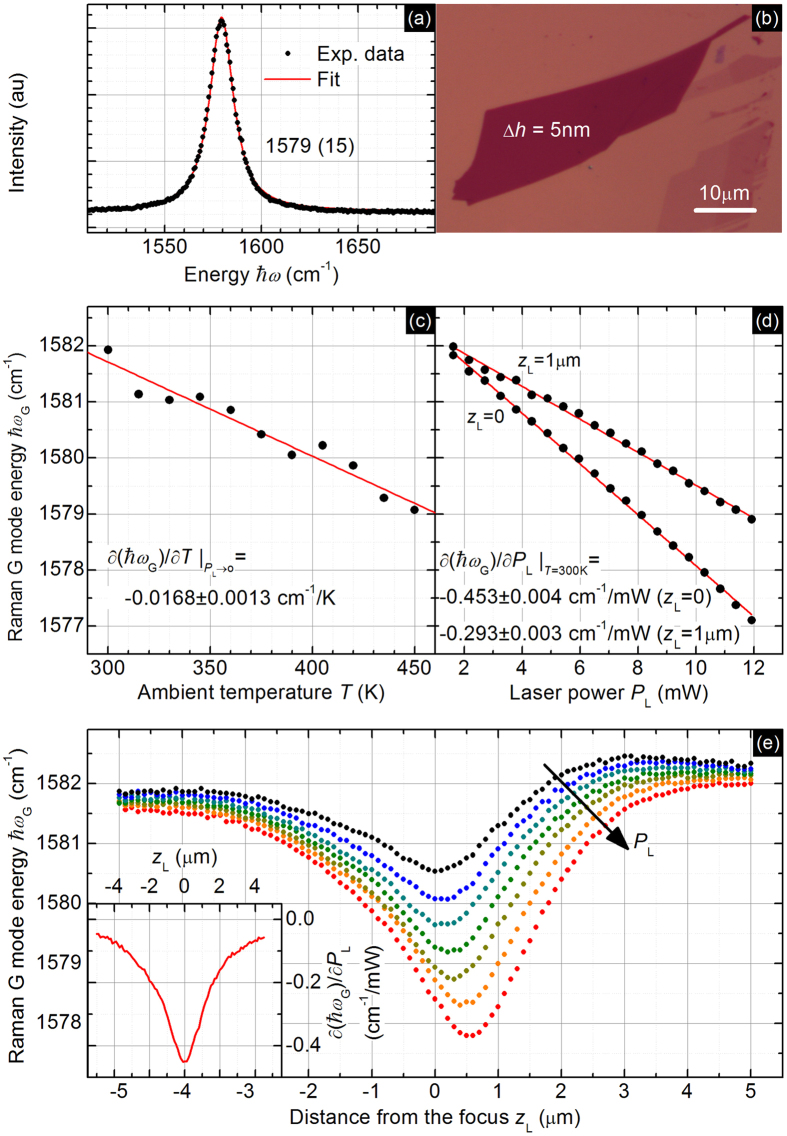
(**a**) Feature in the Raman spectrum, called G mode, characteristic for sp^2^ carbons and illustrating the distribution of the TO + LO phonon energies (tangential and longitudinal optical modes are usually degenerated in the Γ point; therefore, the energy distribution can be fitted with a single Lorentzian function). (**b**) Optical image of a 5-nm thick exfoliated multilayer graphene flake on the Si/SiO_2_ substrate for one of the examined samples. Dependence of the Raman G mode energy from (**c**) temperature taken in the *P*_L_ → 0 limit, (**d**) the power of the laser beam illuminating the sample taken at *T* = 300 K, and (**e**) the distance of the thin film surface from the laser beam focus for seven laser power values (from 5 mW to 11 mW in equal steps). Inset: derivative of the G mode energy over the laser power.

Figure 3a shows an exemplary result of the Raman measurement on the multilayer graphene flake illustrated in the Fig. 3b. The Raman spectrum contains a G mode, one of the typical phonon features of sp^2^ carbons, which has a peak characterized by the position, width and intensity. The first two parameters are temperature dependent and can be used as the thermometers of the averaged local lattice temperature.

In this work, we used the position of the G mode as the temperature indicator because its dependence on the temperature is linear and distinct in the considered temperature range (approximately 300–550 K). Fig. 3c shows the G mode position extrapolated to the limit of zero laser power for different ambient temperature values in the 300–450 K range. The *P* → 0 limit is required because we want to exclude the laser heating that can perturb the value of the local temperature. Simple measurements with low laser power values are, however, unsuitable due to the still observable heating and low signal-to-noise ratio. Therefore, we performed a series of measurements with different laser power values for each ambient temperature value, and we made a linear fit to the experimental data for obtaining the slope and intercept (for each temperature separately). The latter is shown in the Fig. 3c. At the end of the calibration procedure, the value of the derivative of the position with respect to the temperature taken as the slope of the linear fit to the extrapolated data was found to be *χ* = −0.0168 ± 0.0013 cm^−1^/K. Such a value is in good agreement with other works^22^. The given value of the uncertainty comes from the fitting procedure.

Figure 3d shows the dependence of the G mode position versus the laser power for two distances of the sample surface from the laser beam waist, or equivalently, for two beam size values. Four conclusions could be drawn from this picture. First, both dependencies are linear. This is an important issue because it allows for the simple extrapolation of the G mode position to the zero power limit as well as for calculating the derivative with respect to the power 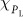 by making a linear fit. Second, the linear dependence of the G mode position both on temperature and laser power allows for the calculation of the temperature increase from the laser power:


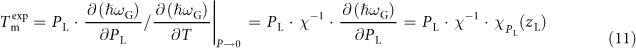


Third, linear dependence on the laser power for two different beam spot size values indicates that there were no significant changes in the thermal properties (no changes in the *κ* and *g* values) of multilayer graphene in the considered laser power range. To prove it, one has to notice that the solution *T*(*r*) of the system of partial differential equations expressed by eqs 7, 8, 9, 10 are directly proportional to *Q* = *α*·*P*_*L*_ (*α* stands for absorption). Therefore, we can substitute 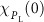 instead of *T*_*m*_(0) or 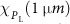 instead of *T*_m_(1 μm) and 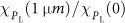 instead of *T*_m_(1 μm)/*T*_m_ in Fig. 2b,d, respectively. Because both derivatives are fixed, they uniquely designate only one pair of *κ* and *g*, *q*.*e*.*d*. Fourth, taking derivatives with respect to the power instead of the G mode position removes unwanted contributions that are not related to the laser heating, *e*.*g*., the instrumental systematic error.

Figure 3e presents the main result of this paper, which is the changes in the averaged temperature increase as a function of the beam size expressed as changes in the G mode position as a function of the distance of the sample surface from the laser beam waist. The slight asymmetry clearly observed in the picture was identified as an instrumental systematic error. To remove this obstacle, we performed our experiment for few laser power values and took the derivative with respect to the *P*_L_, taking into account the small but observable shift of the minimum. The result is presented in the inset of the Fig. 3e. No background can be observed, and the curve is symmetric.

**Figure 4:**
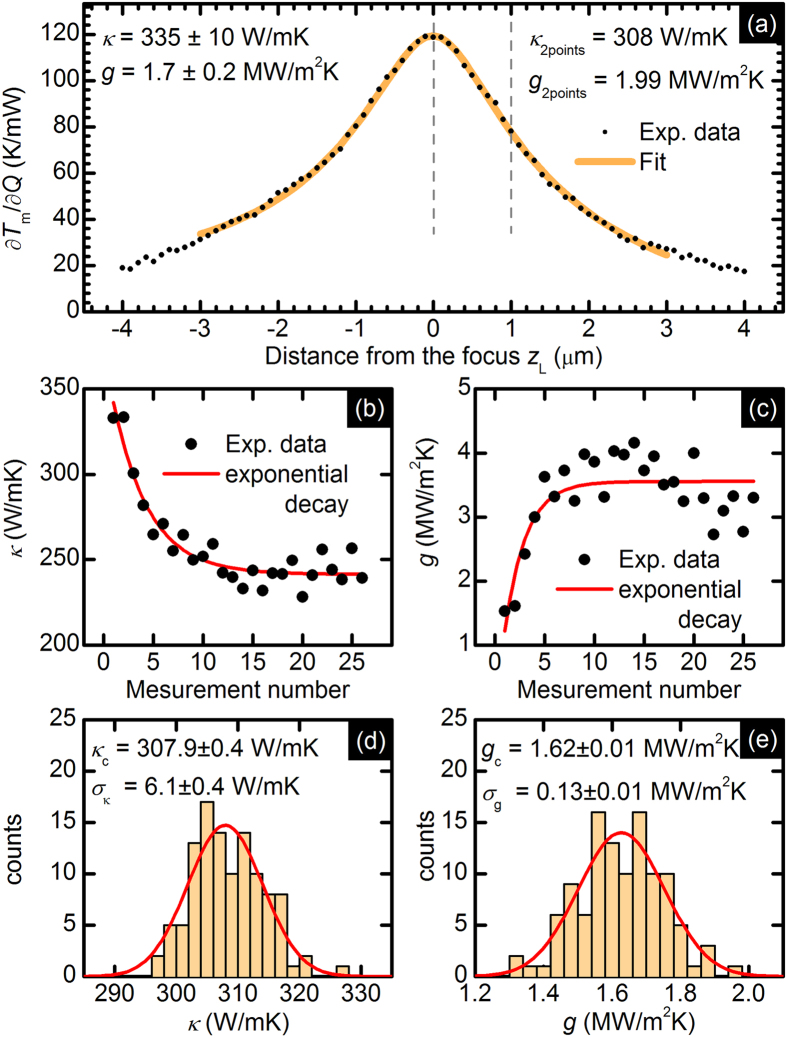
(**a**) Experimental data assisted by the fitted theoretical curve illustrating the derivative of the average temperature increase over the absorbed power versus distance from the focus *z*_L_. Thermal conductivity (**b**) and interface conductance (**c**) values obtained in the first measurements assisted by the fit of the function describing exponential decay (*y*(*x*) = *y*_0_ + *e*^−*x*/*t*^). A rare but possible effect. The histograms of the values of *κ* (d) and *g* (**e**) extracted from 120 consecutive measurements in a typical situation, when sample retains its properties. The red line denotes the fit of the Gaussian function centered at *κ*_c_ or *g*_c_ and characterized by *σ*_κ_ or *σ*_g_. The time of single measurement equals always 120 seconds.

Figure 4a shows the derivative of the local temperature increase with respect to the absorbed power obtained from the derivative of the mode position with respect to the laser power using data from Fig. 3c and the relationship *Q* = *α*·*P*_*L*_, where *α* = 0.225. This operation is the weakest point in our method (as well as in the original opto-thermal method) because it requires the knowledge of the absorption of the 2D material, which is very difficult to find experimentally, and some justified assumptions are indispensable. Here, we consider multilayer graphene as the superposition of single independent atomic sheets^23^. Based on the above, we estimated absorption as *α*_*n+*1_ = *α*_*n*_ + (1 − *α*_*n*_)·*α*_SLG_, *α*_1_ = *α*_SLG_ ≈ 0.0229^24^, where *α*_SLG_ is the absorption of a single layer of graphene and *n* stands for the number of layers (here *n*_max_ = 11 on the basis of the AFM experiment).

Extraction of *κ* and *g* from the experimental data involves a nonlinear fitting procedure, minimizing the *χ*^2^ (chi-squared) value, which is defined as the sum of squared differences between ∂*T*_m_/∂*Q*(*z*_L_) and *T*_m_(*κ*, *g*, *z*_L_, *r*_0_, *NA*, *Q*) with previously found values describing the laser beam, *r*_0_ and *NA* and for *Q* = 1. Similar, but simpler approach for *κ* extraction was proposed by S. Ghosh, who adjusted the *κ* value in the theoretical model to obtain equal theoretical and experimental temperature increase values^6^. The results of the above procedure are shown in Fig. 4a. Excellent agreement between experimental data and the theoretical curve is achieved for *κ* = 335 ± 10 W/mK and *g* = 1.7 ± 0.2 MW/m^2^K. Both uncertainties come from the fitting procedure and do not include systematic errors from the uncertainty of *Q*, *r*_0_ and *NA*. For comparison, we calculated the thermal conductivity and interface conductance basing on results from Fig. 3d, especially the derivatives calculated for two different distances of the sample surface from the beam waist. The obtained results, *κ* = 308 W/mK and *g* = 1.99 MW/m^2^K, are in good agreement with the results from the new method; however, it should be emphasized that we determined the exact focus position from the ∂*T*_m_/∂*Q*(*z*_L_) measurements.

Now, we turn our attention to the issue of the accuracy and repeatability of our method. Two different aspects should be discussed. The first is the change of the *κ* and *g* in time due to any changes in the 2D material properties under laser irradiation. The changes in the structural properties of graphene monolayers due to laser irradiation were previously reported^19^,^20^,^21^, which is why we use multi-layer structures instead of single-layer structures. In most cases, the values of *κ* and *g* obtained in the first measurements do not differ from the values obtained in further measurements (in the uncertainty limit). However, a situation similar to the one illustrated in Fig. 4b,c rarely might take place. In Fig. 4b, a clear and distinct drop in the value of the thermal conductivity is observed in spite of the presence of statistical noise. After a few measurements, the value of *κ* stabilizes and does not further change significantly in time. It is important that there is no D mode indicating the presence of defects. A similar situation can be observed in Fig. 4c, where an initial increase in the interface conductance is observed. Simultaneous drop in the value of the thermal conductivity and increase of the value of the interface conductance, in our opinion, suggest increased adhesion as the most possible explanation of observed effect. Transient heating of substrate has been excluded.

The second issue is the statistical uncertainty of the values of *κ* and *g*. To examine this uncertainty, we performed a series of identical 120 consecutive measurements. The distribution of the extracted values of *κ* and *g* is shown in Fig. 4d,e. The statistical uncertainty of the thermal conductivity equals 2%; the statistical uncertainty of the interface conductance equals 8%. The above values are extremely important because they clearly define the accuracy of the presented method. The systematic uncertainty is not considered here because for comparison studies, the statistical uncertainty is important. Low statistical uncertainty and easy automation enables the use of our method for new types of studies in the micro-scale, *e*.*g*., thermal conductivity mapping, which has not been reported yet.

**Figure 5:**
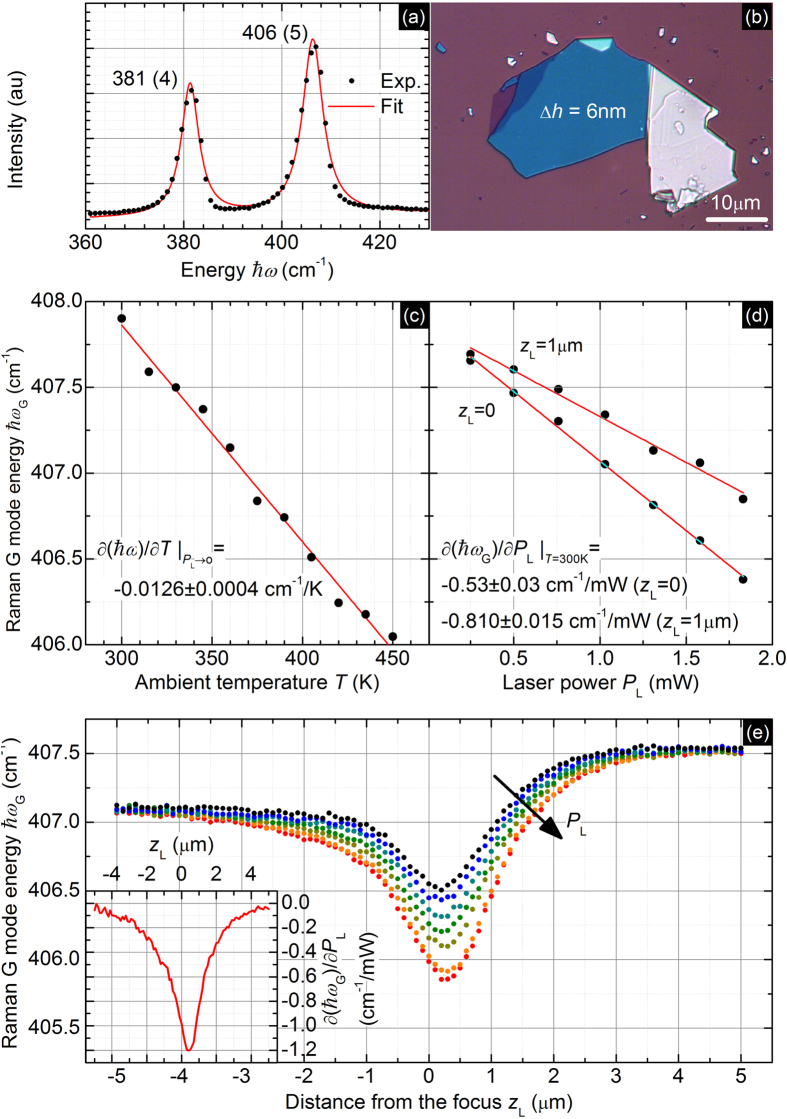
(**a**) An exemplary spectrum of the multilayer MoS_2_ consisting of two peaks, denoted as E_2g_ (the left one) and A_1g_ (the right one). (**b**) Optical image of 6-nm thick exfoliated multilayer MoS_2_ flake on the Si/SiO_2_ substrate – one of the examined samples. Dependence of the Raman A_1g_ mode energy from (**c**) the temperature taken in the *P*_L_ → 0 limit, (**d**) power of the laser beam illuminating the sample taken at *T* = 300 K, and (**e**) distance of the thin film surface from the laser beam focus for seven laser power values (*P* = 1.08, 1.19, 1.29, 1.39, 1.48, 1.58, and 1.69 mW). Inset: derivative of the G mode energy with respect to the laser power.

Figure 5a shows an exemplary result of the Raman measurement on the multilayer MoS_2_ flake illustrated in Fig. 5b. The Raman spectrum contains two modes denoted as E_2g_ at 381 cm^−1^ and A_1g_ at 406 cm^−1^. As the thermometer, we use the position of the A_1g_ mode because of higher sensitivity on the temperature change. In the Fig. 5c, we show the dependence of the position of the A_1g_ mode on the temperature in the limit of zero power, whereas in Fig. 5d, we present the dependence of the position on the laser power at ambient temperature. The derivative of the A_1g_ mode position with respect to temperature is *χ* = −0.0126 ± 0.0004 cm^−1^/K, which is in good agreement with other studies^25^. The dependence of the mode position versus laser power is linear, which is a prerequisite for the applicability of the method.

**Figure 6:**
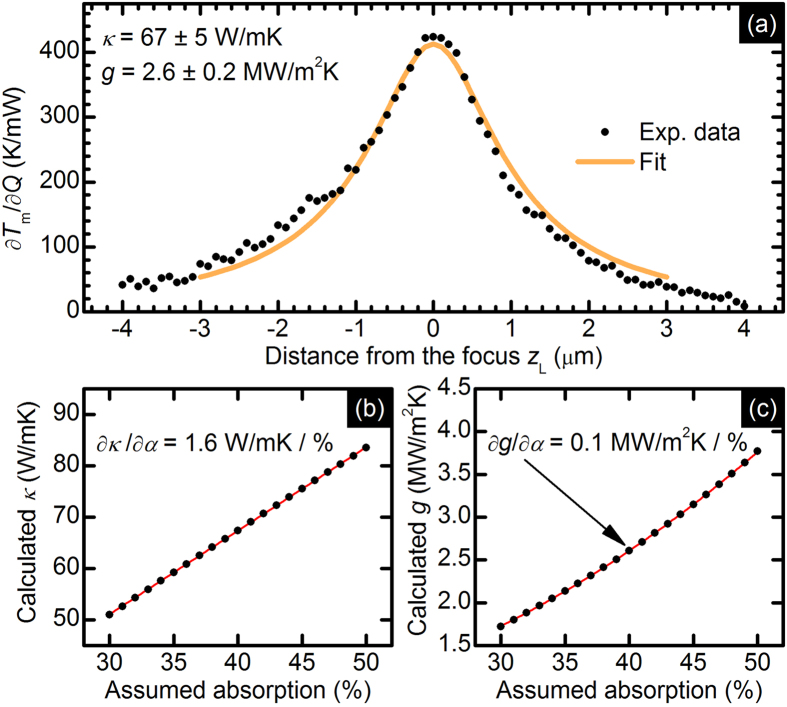
(**a**) Experimental data assisted by the fitted theoretical curve, illustrating the derivative of the average temperature increase over the absorbed power versus distance from the focus *z*_L_. The dependence of the calculated thermal conductivity *κ* (**b**) and interface conductance *g* (**c**) on the assumed absorption.

Figure 5e presents the dependence of the Raman mode position (local temperature increase) on the distance from the focus position (heat source size). Large asymmetry originates from the systematic instrumental error and is eliminated by taking the derivative of the mode position with respect to the laser power (inset). The effect of the transformation to the derivative of the local temperature increase with respect to the heating power performed using the assumption on the absorption level (40%) is shown in Fig. 6a. The theoretical fit follows the experimental data well. The obtained results *κ* = 67 ± 5 W/mK and *g* = 2.6 ± 0.2 MW/m^2^K are in a good agreement with literature reports based on Raman spectroscopy^26^,^27^ and electrical thermometry^28^, but differ from the reports based on MOKE^11^. One of the possible explanations of this discrepancy is the contamination of the samples by the organic residues coming from polymer-film-assisted transfer process, that can significantly suppress the measured thermal conductivity^29^.

Because the absorption measurements of thin flakes require specially prepared substrates (see, *e*.*g*., ref. 3) and the estimation methods have been not yet established, we decided to calculate the thermal conductivity (Fig. 6b) and total interface conductance (Fig. 6c) values for different absorption levels in the range of 30–50%^30^. Such an approach has an additional advantage because it shows the sensitivity of the extracted parameters on the assumed absorption level (uncertainty).

In summary, we demonstrated a method for the high accuracy determination of both *κ* and *g* of supported 2D materials. We exploited the property of the laser beam, a precisely defined power distribution in all directions, to develop a new approach of data acquisition and theoretical analysis in the opto-thermal method, successfully eliminating its weak points. Finally, we demonstrated that our method is applicable for two 2D materials that are characterized by different thermal properties.

## Methods

All samples were produced by the exfoliation method from bulk crystals. The substrate we used had a p-doped Si wafer covered by the thermally grown SiO_2_ dielectric layer with a 285-nm thickness. The AFM pictures were collected using the NT-MDT Ntegra system. The Raman measurements were performed using a Renishaw inVia Raman spectrometer equipped with a motorized XYZ stage with 100-nm resolution. All spectra were collected using a 514-nm laser line in one spectral window and 3000 lines/mm grating, and a CCD camera was used to detect inelastically scattered photons. To exclude any symmetry-based phenomena, we used circularly polarized light. The Raman modes were always fitted with one Lorentzian function and linear background. The laser power value was measured using an Ophir Nova II system with photodiode sensor PD-300.

## Additional Information

**How to cite this article**: Judek, J. *et al.* High accuracy determination of the thermal properties of supported 2D materials. *Sci. Rep.* **5**, 12422; doi: 10.1038/srep12422 (2015).
